# Importance of Experimental Environmental Conditions in Estimating Risks and Associated Uncertainty of Transgenic Fish Prior to Entry into Nature

**DOI:** 10.1038/s41598-018-35826-1

**Published:** 2019-01-23

**Authors:** Wendy E. Vandersteen, Rosalind Leggatt, L. Fredrik Sundström, Robert H. Devlin

**Affiliations:** 1 Department of Fisheries and Oceans, 4160 Marine Dr., West Vancouver, BC V7V 1N6 Canada; 2 Present Address: Miracle Springs Inc., 12443 Stave Lake Rd., Mission, BC V2V 0A6 Canada; 3 Present Address: Uppsala University, Biology Education Centre, Norbyvägen 14, SE-752 36 Uppsala, Sweden

## Abstract

Salmonids show a high degree of phenotypic plasticity that can differ among genotypes, and this variation is one of the major factors contributing to uncertainty in extrapolating laboratory-based risk assessment data to nature. Many studies have examined the relative growth and survival of transgenic and non-transgenic salmonids, and the results have been highly variable due to genotype × environment interactions. The relative survival of fast- and slow-growing strains can reverse depending on the environment, but it is not clear which specific environmental characteristics are driving these responses. To address this question, two experiments were designed where environmental conditions were varied to investigate the contribution of rearing density, food amount, food type, habitat complexity, and risk of predation on relative growth and survival of fast-growing transgenic and slow-growing wild-type coho salmon. The first experiment altered density (high vs. low) and food amount (high vs. low). Density impacted the relative growth of the genotypes, where transgenic fish grew more than non-transgenic fish in low density streams, regardless of food level. Density also affected survival, with high density causing increased mortality for both genotypes, but the mortality of transgenic relative to non-transgenic fish was lower within the high-density streams, regardless of food level. The second experiment altered habitat complexity (simple vs. complex), food type (artificial vs. natural), amount of food (normal vs. satiation), and risk of predation (present vs. absent). Results from this experiment showed that genotype affected growth and survival, but genotype effects were modulated by one or more environmental factors. The effect of genotype on survival was influenced by all examined environmental factors, such that no predictable trend in relative survival of transgenic versus non-transgenic fry emerged. This was primarily due to variations in survival of non-transgenic fish under different environmental conditions (non-transgenic fry had highest survival in hatchery conditions, and lowest survival in complex conditions with natural food fed at a normal level with or without predators). Transgenic fry survival was only significantly influenced by predator presence. The effects of genotype on mass and length were significantly modulated by food type only. Transgenic fry were able to gain a large size advantage over non-transgenic fish when fed artificial food under all habitat types. These experiments support the observations of dynamic responses in growth and survival depending on the environment, and demonstrate the challenge of applying laboratory-based experiments to risk assessment in nature.

## Introduction

With approval of growth-hormone transgenic Atlantic salmon (*Salmo salar*) commercial production for Canada and the United States markets, and recent sales of unlabeled fish in Canada, there is renewed discussion within the aquaculture industry on the relevance and use of transgenic fish. As attempts are made to move the production of salmon from the ocean to land with the use of recirculating aquatic systems, the option of sterile, all-female, faster-growing strains of salmon with increased feed conversion efficiency may become appealing to some developers exploring the feasibility of a land-based aquaculture industry. Although not currently approved, should production scenarios emerge where transgenic fish strains are reared in conditions where they could escape to nature, it will be imperative to fully understand major factors affecting potential environmental impacts, under a range of environmental conditions, for use in formal risk assessments. Understanding phenotypic effects of a transgene that influence fitness (survival and reproductive success) and consequences to ecosystem components are key requirements to allow potential impacts to be robustly predicted for use in environmental assessments^1–6^.

Obtaining empirical data from experiments in nature would minimize uncertainty in ecological risk assessments of fast-growing transgenic fish. However, the eradication of transgenic fish from the aquatic environment would be essentially impossible^7^ and could be detrimental if negative consequences occurred. Instead, risk assessments have been based on assessments of potential ecological impacts in laboratory settings^5^, an approach that increases uncertainty^4,6^. This uncertainty could be reduced by a deeper understanding of the specific environmental characteristics influencing variability in assessment data^5,8^.

One of the major factors contributing to uncertainty in extrapolating laboratory-based risk assessments to nature is that many species of fish, including salmonids, show a high degree of phenotypic plasticity^9^. The phenotype of an individual is influenced not only by its genotype but also by environmental conditions, and distinct genotypes can respond to different environments in dissimilar ways (genotype × environment interactions). There have been many studies examining the growth and survival of fast-growing transgenic salmonids relative to slow-growing wild conspecifics under different environmental conditions. The results of these studies have been highly variable due to such genotype × environment interactions (see Table 1 in^4^, for example). When reared under typical hatchery conditions with excess feed and simple tank environments, different species of transgenic salmonids outgrow non-transgenic strains^10–19^. When reared under varying simulated natural conditions, responses are mixed: the transgenic fish may still demonstrate increased growth (although of lesser magnitude than hatchery conditions^13,17,20–24^, show no difference in growth^21,25–28^, or display reduced growth^20,23,24^ relative to non-transgenic fish. Similarly, transgenic salmonids have shown higher mortality relative to non-transgenic salmonids in some experiments^20,24–26^ but other studies found no difference in survival^13,23–28^ or even increased survival^13,21,23^.

**Table 1:** *P* values, Chi-squared values and rank orders (given as *P* (χ^2^, rank)) for factor effects, factor interactions, and individual differences in genotype (T = transgenic; NT = non-transgenic) within treatment for the density/food amount experiment: HDHF = high density, high food; HDLF = high density, low food; LDHF = low density, high food; LDLF = low density, low food.

	Mass	Length	CF	Survival
*Factor and Interaction effects*				
Genotype	*0.001 (10.2)*	*<0.001 (18.4)*	*0.826 (0.0)*	*<0.001 (66.4)*
Density	*<0.001 (27.7)*	*<0.001 (29.5)*	*0.807 (0.1)*	*<0.001 (63.6)*
Food	*0.031 (4.7)*	*0.026 (4.9)*	*0.344 (0.9)*	<0.001 (20.0)
Genotype × Density	<0.001 (13.1, T>NT when density Low only)	<0.001 (45.4, T>NT when density Low only)	0.582 (0.3)	0.013 (6.2, T>NT when density High only)
Genotype × Food	0.591 (0.3)	0.283 (1.2)	*0.058 (3.6)*	0.410 (0.7)
Density × Food	<0.001 (17.3)	<0.001 (20.5)	*0.160 (2.0)*	0.133 (2.2)
Genotype × Density × Food	0.856 (0.0)	0.550 (0.4)	0.034 (4.5)	0.694 (0.2)
*Genotype effects within treatment*				
HDHF	0.21	0.26	0.94	0.013 (T>NT)
HDLF	0.3	0.43	0.13	0.002 (T>NT)
LDHF	<0.001 (T>NT)	0.001 (T>NT)	0.66	1
LDLF	0.013 (T>NT)	0.015 (T>NT)	0.16	1

Variables examined are mass (g), length (cm), condition factor (CF = g / cm^3^ * 100), and survival at the end of the experiment. Italic numbers indicate presence of significant interaction effects so these individual factor effects should not be considered.

It is clear that relative survival of fast- and slow-growing strains can vary (including reverse) depending on the environment, but the specific environmental characteristics driving these trends and uncertainties are not fully known. Some factors identified to reverse relative growth and survival trends of transgenic and wild-type strains include prior rearing conditions^26^ and interactions among food level and presence or timing of predator exposure^23,29^. Better understanding of the relative role and impact of different environmental characteristics will help to reduce the uncertainty involved with ecological risk assessments. To address this question, we performed two experiments where environmental conditions were varied to investigate the contribution of rearing density, food amount, food type, habitat complexity, and risk of predation on relative growth and survival of fast-growing transgenic and slow-growing wild-type coho salmon (*Oncorhynchus kisutch*).

## Materials and Methods

All experiments were conducted at Fisheries and Oceans Canada (DFO) research facility in West Vancouver, Canada. This facility is specially designed for research on assessing potential ecological impact of transgenic fish and has multiple containment systems to prevent the escape of fish into the natural environment. Experiments were conducted in compliance with Canadian Council of Animal Care Guidelines under permit from the DFO Pacific Region Animal Care Committee.

### Fish

Fast-growing transgenic coho salmon were initially produced by microinjecting the OnMTGH1 growth hormone gene construct (growth hormone I gene driven by the metallothionein promoter, both from sockeye salmon) into eggs from wild-caught parents from the Chehalis River in southwestern British Columbia^19^. A stable and characterized strain of the fast-growing transgenic fish was developed through crosses with wild-caught fish from the Chehalis River at each generation^19^; this transgenic strain therefore contains the same genetic background as the wild-type strain except for the presence of the OnMTGH1 transgene. The transgenic fish (strain M77) used for the present experiments were F_9_ and F_10_ generation offspring of transgenic males and non-transgenic Chehalis River females.

### Density and amount of food

On 4 March 2009, half of the eggs from five wild-caught females were fertilized with milt from five laboratory-reared males homozygous for the transgene (single-pair crosses); the other half of the eggs from the five females were fertilized with milt from five wild-caught males from the Chehalis River. The eggs were reared in Heath trays using well water chilled to between 4-5 °C. On 18 September 2009, the fry were placed into eight semi-natural stream tanks (5 × 1 × 0.4 m) enriched with a gravel bottom, a large log, two plants, and four large rocks. A slow water current was maintained within the stream tanks by supplying natural creek water (10 L/min) at one end, and allowing it to flow out the other end. The experimental tanks received either 150 non-transgenic and 150 transgenic fry (300 fry total, 0.15 fish/L, 60 fish/m;^2^ High Density, HD) or 15 non-transgenic and 15 transgenic fry (0.015 fish/L, 60 fish/m;^2^ Low Density, LD). Half of the tanks were fed a relatively high amount of food (High Food, HF) that was double the amount and frequency of food fed to the other tanks (Low Food, LF) resulting in a x4 difference in amount of food provided. Food consisted of live or frozen brine shrimp, frozen bloodworms, frozen *Mysis*, live crickets, and any live food that was carried in through the creek water inflow^17^. In total, there were two tanks of each possible combination: HDHF, HDLF, LDHF, LDLF, and genotype, density, food level, and their interactions, were assessed (Table 1). On 11 May 2010 all fish were captured from the stream tanks, measured in length and mass, and adipose fin-clipped for genotyping using a PCR test specific for the transgene^30^.

### Habitat complexity, food type, amount of food, and risk of predation

On 19 February 2010, eggs from nine wild-caught females were pooled and separated into seven aliquots. Each aliquot was fertilized with milt from a hatchery-reared male hemizygous for the transgene; therefore, the population consisted of 50% transgenic fry and 50% non-transgenic siblings. A random sample of 96 fry genotyped by PCR analysis detected 47 transgenic, 48 non-transgenic, and 1 unknown fry, confirming the expectation of equal proportions of transgenic and non-transgenic fish based on Mendelian segregation of the transgene. These fry were transferred to 3000 L tanks on 7 May 2010 and fed lightly until they were placed into 12 experimental stream tanks (same as above) on 20 May 2010 (100 fish total per tank, 0.05 fish/L). The experimental streams (5 × 1 × 0.4 m) were supplied with a slow water current (10 L/min) maintained by natural (surface) creek water entering at one end and allowing it to flow out the other end. For this experiment, four environmental characteristics were altered: habitat complexity (simple or complex), food source (natural or artificial), food amount (satiation or normal), and risk of predation (present or absent). Table 2 summarizes the characteristics of each experimental tank. On 3 June 2010 one cutthroat trout was added to tanks 4 and 10 as predators. Two control tanks of 100 fish per tank were maintained under hatchery conditions with creek water (in 15 L netted buckets within 200 L tanks up to a density of 6.7 fish/L in the bucket and then released into the 200 L at 0.5 fish/L in the tank). The fish remained within the tanks for one year (until May 2011) when they were captured, weighed, measured, and adipose fin-clipped for genotyping.

**Table 2:** *P* values and genotype rank orders for mass, length, and survival under different environmental conditions. Complex habitats consisted of a gravel bottom, a large log, two plants, and four large rocks whereas simple habitats were bare tanks. Natural food (Nat) included live or frozen brine shrimp, frozen bloodworms, frozen *Mysis* and live crickets, and artificial food (Art) consisted of commercial pellets (Skretting Canada Ltd.).

Treatment	Habitat	Food		Predator	Genotype difference, *P* value		
		Type	Amount		Mass	Length	Survival
A	Complex	Nat	Normal	No	=, 0.55	=, 0.51	T>NT, 0.042
B	Complex	Art	Satiation	No	T>NT, 0.004	T>NT,<0.001	NT>T, 0.028
C	Simple	Nat	Normal	No	=, 0.22	=, 0.21	=, 0.58
D	Complex	Nat	Normal	Yes	n/a	n/a	=, 0.28
E	Complex	Nat	Satiation	No	T>NT, 0.0401	=, 0.054	=, 0.082
F	Simple	Art	Satiation	No	T>NT, 0.006	T>NT, 0.01	=, 0.79
Hatchery	Hatchery	Art	Satiation	No	T>NT, 0.002	T>NT, 0.003	NT>T, 0.037

A normal amount of food was half the satiation ration; the satiation ration was approximated by feeding the fish until a small amount of excess food remained on the bottom of the tank. T = transgenic; NT = non-transgenic.

### Statistical Analysis

Statistical analyses were performed using R 3.2.1^31^. Differences in survival and size between genotypes under varying environmental conditions were tested with generalized linear mixed models using the lme4 package^32^. Genotype, environmental conditions (see below for specific conditions), and their interactions, were included as fixed factors, and genotype was crossed with the random factor stream tank. Variables were modelled using the normal distribution and identity link (log-mass, fork length and condition factor) or binomial distribution and log-link function (survival)^32^.

For the first experiment examining density and food level, variables were modeled as [(g)lmer (response variable ~ Genotype*Density*Food Amount + (1|Stream/Genotype))]. For the second experiment examining additional stream conditions, size variables (excluding Hatchery data) were modelled as [lmer (variable ~ Genotype*(Habitat * Food Type + Food Amount) + (1|Stream/Genotype))]. Predation was excluded from the size models as transgenic fish had 0% survival in one predation tank, resulting in n = 1 treatment tank for this group. To include data from hatchery-reared fish, the model was run where each treatment combination was considered a unique level as [lmer (response variable ~ Genotype*Treatment + (1|Tank/Genotype))]. Survival data was modelled as per size data above except with the glmer function, including a random variable (1/obs) to account for over-dispersion, and optimizer control bobyqa [e.g. control = glmerControl(optimizer = ‘bobyqa’); the bobyqa algorithm solves the problem using a trust region method that forms quadratic models by interpolation]to ensure model convergence, and predation was included as a factor.

Significance of the model outputs were evaluated using the Anova() function in the car package with test type 3^33^; e.g. Anova(model, contrasts = list(topic = contr.sum, sys = contr.sum), type =3)]. Holm-Bonferroni *post-hoc* comparisons between genotypes within treatment or treatment within genotype were evaluated using predicted values based on above models (predict() function in lme4 package). Data are given as mean of genotype within treatment tanks ± standard error of the mean, calculated from the predicted values from the model outputs.

## Results

### Density and amount of food

**Figure 1:**
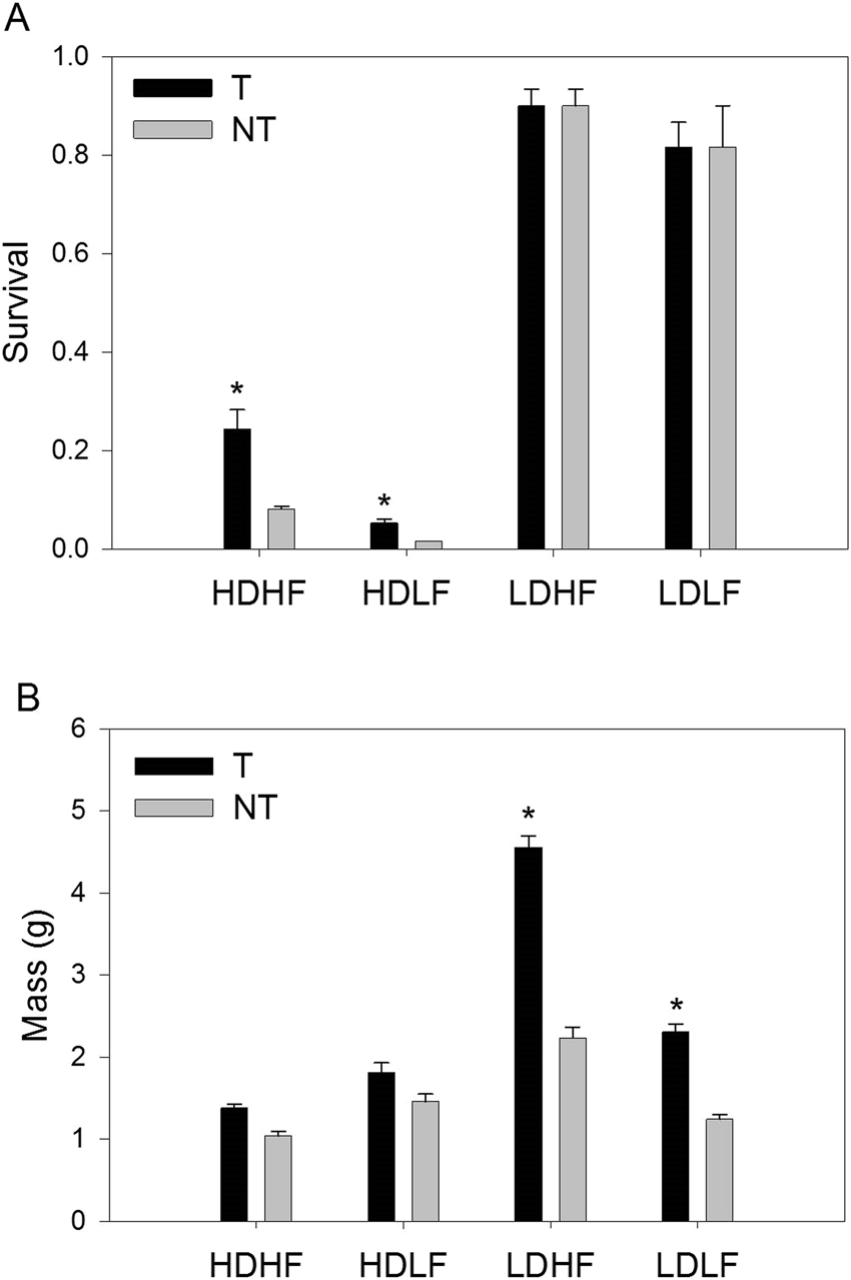
Survival as proportion (**A**) and size (**B**) of transgenic (T) and non-transgenic (NT) fish reared under semi-natural conditions of high density (HD), low density (LD), high food (HF) and low food (LF). Error bars represent SEM. * indicates significant differences between T and NT within treatment.

At the end of the growth trial, there was a significant interaction between genotype and density for mass, length and survival (Table 1). Density impacted the relative growth of both genotypes, with transgenic fish having a larger increase in mass relative to non-transgenic fish in low-density streams, regardless of food level (Fig. 1). Density also had an overall effect on survival with fish at high density having much poorer survival for both genotypes than at low density. However, the survival of transgenic relative to non-transgenic fish was higher within the high density streams, regardless of food level (Fig. 1). Food level did not influence the rank or survival relationship between transgenic and non-transgenic fish. There was a significant three-way interaction among genotype, food level and density on condition factor, but no differences between transgenic and non-transgenic fish within any treatment combination (Table 1).

### Habitat complexity, food type, amount of food, and risk of predation

**Figure 2:**
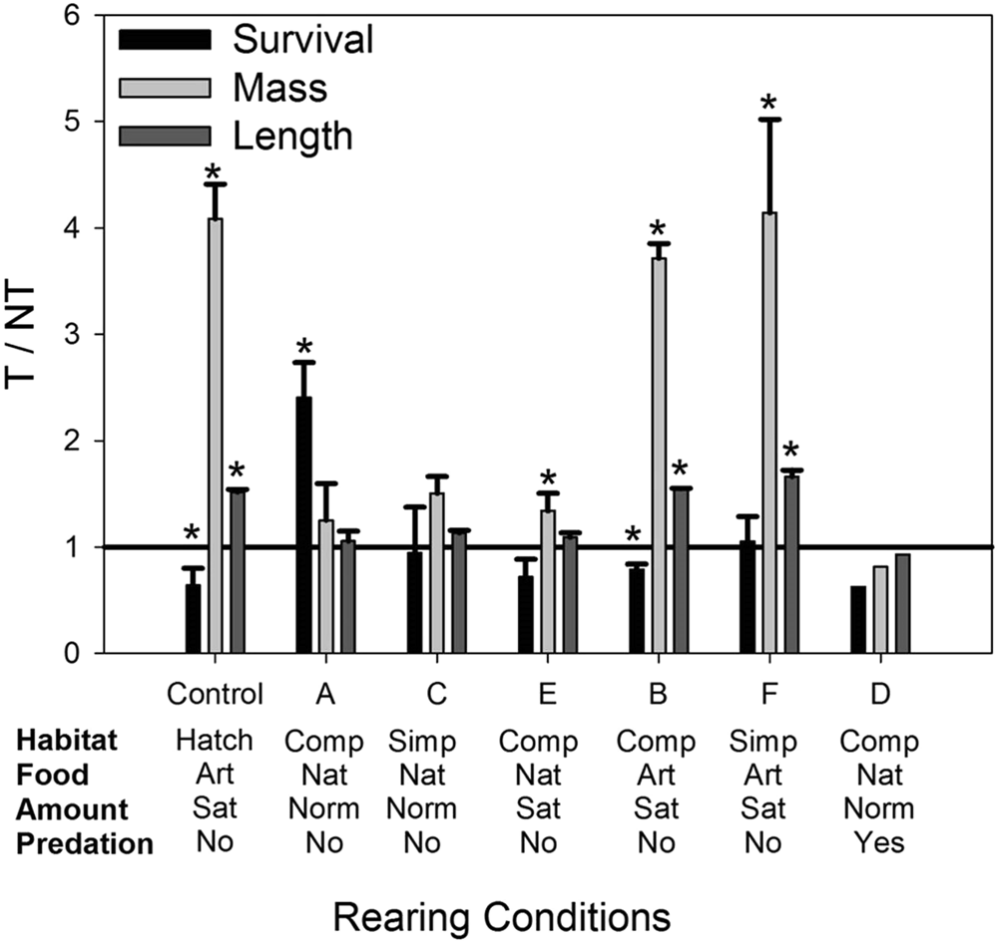
Relative (T/NT) survival, mass, and length of growth hormone transgenic (T) or non-transgenic (NT) coho salmon fry reared under different environmental conditions. Habitats varied from standard hatchery conditions (Hatch), complex habitats (Comp) consisted of stream tanks with gravel bottom, a large log, two plants, and four large rocks, or simple habitats (Simp) were bare stream tanks. Natural food (Nat) included live or frozen brine shrimp, frozen bloodworms, frozen *Mysis*, and live crickets, and artificial food (Art) consisted of commercial pellets (Skretting Canada Ltd.). A satiation food amount (Sat) was approximated by feeding the fish in the tanks until excess food remained on the bottom of the tank, and normal amount of food (Norm) was half the satiation ration. Bars above the horizontal line indicate T>NT, below the line indicate NT>T, and * indicates these differences are statistically significant.

**Figure 3:**
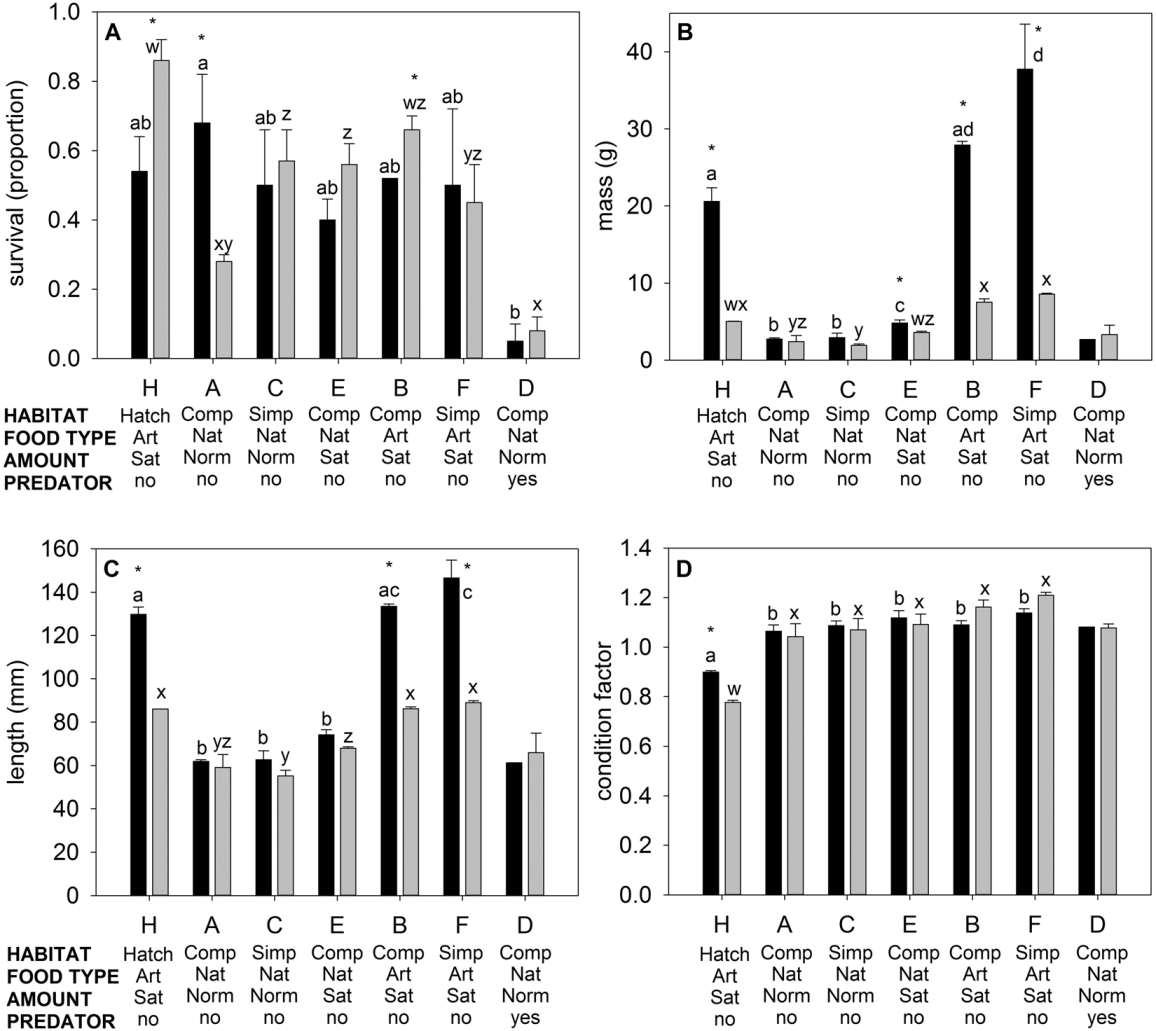
Survival, (**A**), mass (**B**), length (**C**), and condition factor (**D**) of growth hormone transgenic (black bars) or non-transgenic (grey bars) coho salmon fry reared under different environmental conditions. Habitats varied from standard hatchery conditions (Hatch), complex habitats (Comp) consisting of stream tanks with gravel bottom, a large log, two plants, and four large rocks, or simple habitats (Simp) consisting of bare stream tanks. Natural food (Nat) included live or frozen brine shrimp, frozen bloodworms, frozen *Mysis*, and live crickets, and artificial food (Art) consisted of commercial pellets (Skretting). A satiation food amount (Sat) was approximated by feeding the fish in the tanks until excess food remained on the bottom of the tank, and a normal amount of food (Norm) was half the satiation ration. *indicates significant difference between transgenic and non-transgenic fish within treatment, and letters indicate significant differences among treatments within genotype, i.e. (a,b,c,d) within transgenic fish and (w,x,y,z) within non-transgenic fish.

At the end of the environmental conditions trial, although there was a significant effect of genotype on size (mass and length) and survival, genotype effects on all variables were significantly modulated by one or more environmental factors (see Tables 2 and 3 for statistical summaries). For survival, there was no consistent trend in relative survival of transgenic versus non-transgenic fry due to the effects of environmental factors (see Fig. 2 for relative responses of T relative to NT, and Fig. 3 for absolute measures). Transgenic fry had lower survival than non-transgenic fry when fed artificial food under both hatchery conditions and complex stream-tank conditions (treatments Hatchery and B), but equal survival when fed artificial food in simple stream-tank conditions (treatment F, see Fig. 2 for relative survival and size). In contrast, transgenic fry had higher survival with natural food fed at a normal level in a complex habitat, and no predators (treatment A), but equal survival to non-transgenic fry in all other conditions when fed natural food. The inconsistent effects of genotype on relative survival were primarily due to variations in survival of non-transgenic fish under different environmental conditions (i.e. non-transgenic fry had highest survival in hatchery conditions, and lowest survival in complex conditions with natural food fed at a normal level with or without predators), as transgenic fry survival was only strongly influenced by predator presence (see Fig. 3A).

**Table 3:** *P* values and Chi-squared values (given as *P* (χ^2^)) for factor and interaction effects for size and survival measurements under simple or complex Habitat, natural or artificial Food, normal or satiation food Amount, and presence or absence of Predators in stream tanks, as well overall treatment interaction and factor effects including hatchery environment.

Interaction/Factor	Mass	Length	Condition	Survival
*Stream tanks only*				
Genotype × Habitat × Food	0.995 (0.0)	0.514 (0.4)	0.898 (0.0)	<0.001* (11.2)
Genotype × Habitat	0.389 (0.7)	0.093 (2.8)	0.984 (0.0)	*0.194 (1.8)*
Genotype × Food	<0.001* (19.5)	<0.001* (58.3)	0.027* (4.9)	*0.915 (0.0)*
Genotype × Amount	0.624 (0.2)	0.576 (0.3)	0.914 (0.0)	<0.001* (15.8)
Genotype × Predator	n/a	n/a	n/a	0.004* (8.2)
Habitat × Food	0.294 (1.1)	0.086 (2.9)	0.645 (0.2)	*0.002 (9.4)*
Genotype	<0.001* (17.6)	<0.001* (42.8)	*0.171 (1.9)*	0.014* (6.1)
Habitat	0.106 (2.6)	0.013* (6.2)	0.273 (1.2)	*0.071 (3.2)*
Food	<0.001* (75.1)	<0.001* (148)	*0.526 (0.4)*	*0.380 (0.8)*
Amount	0.002* (9.6)	0.011* (6.4)	0.218 (1.5)	0.014* (6.0)
Predator	n/a	n/a	n/a	0.008* (7.0)
*Hatchery* + *Stream tanks*				
Genotype × Treatment	<0.001* (88.8)	<0.001* (205)	<0.001* (32.8)	<0.001* (14.4)
Genotype	*0.236 (1.4)*	*0.459 (0.5)*	*0.451 (0.6)*	*<0.001 (65.4)*
Treatment	*<0.001 (446)*	*<0.001 (725)*	*<0.001 (45.2)*	*<0.001* ^57^

Predator tanks were not included in analysis of size variables due to 100% mortality of Transgenic fry in one predator tank resulting in n = 1 for that treatment/genotype group. * indicates significant effect, and italic text indicates higher level interaction effect so that factor effects should not be considered in isolation.

The effects of genotype on mass and length were significantly modulated by food type only. Transgenic fry were able to gain a large size advantage over non-transgenic fish when fed artificial food under all habitat types (see Fig. 2). However, when fed natural food items, transgenic fry were only able to obtain greater mass than non-transgenic fish when fed to satiation in a complex habitat – in all other conditions when provided with natural food, transgenic and non-transgenic fish did not significantly differ in size. The relative size differences of the two genotypes under alternative food sources were primarily due to a much stronger positive effect of artificial food relative to natural food on transgenic fry size than that observed for non-transgenic fry (e.g. 6-fold versus 2-fold increase in weight respectively, see Fig. 3B,C). Size (both length and weight) of either genotype was not significantly affected by habitat type when other factors were equal (treatments A versus C, or B versus F, see Fig. 3B,C), and increasing natural food amount only increased mass of transgenic fish (treatments A vs. E). While there were some significant factor and interaction effects on condition factor (Table 3), there were few differences other than overall greater condition factor for both genotypes in stream tanks vs. hatchery conditions, and transgenic fry had greater condition factor than non-transgenic fry in hatchery conditions only (see Fig. 3D).

## Discussion

**Figure 4:**
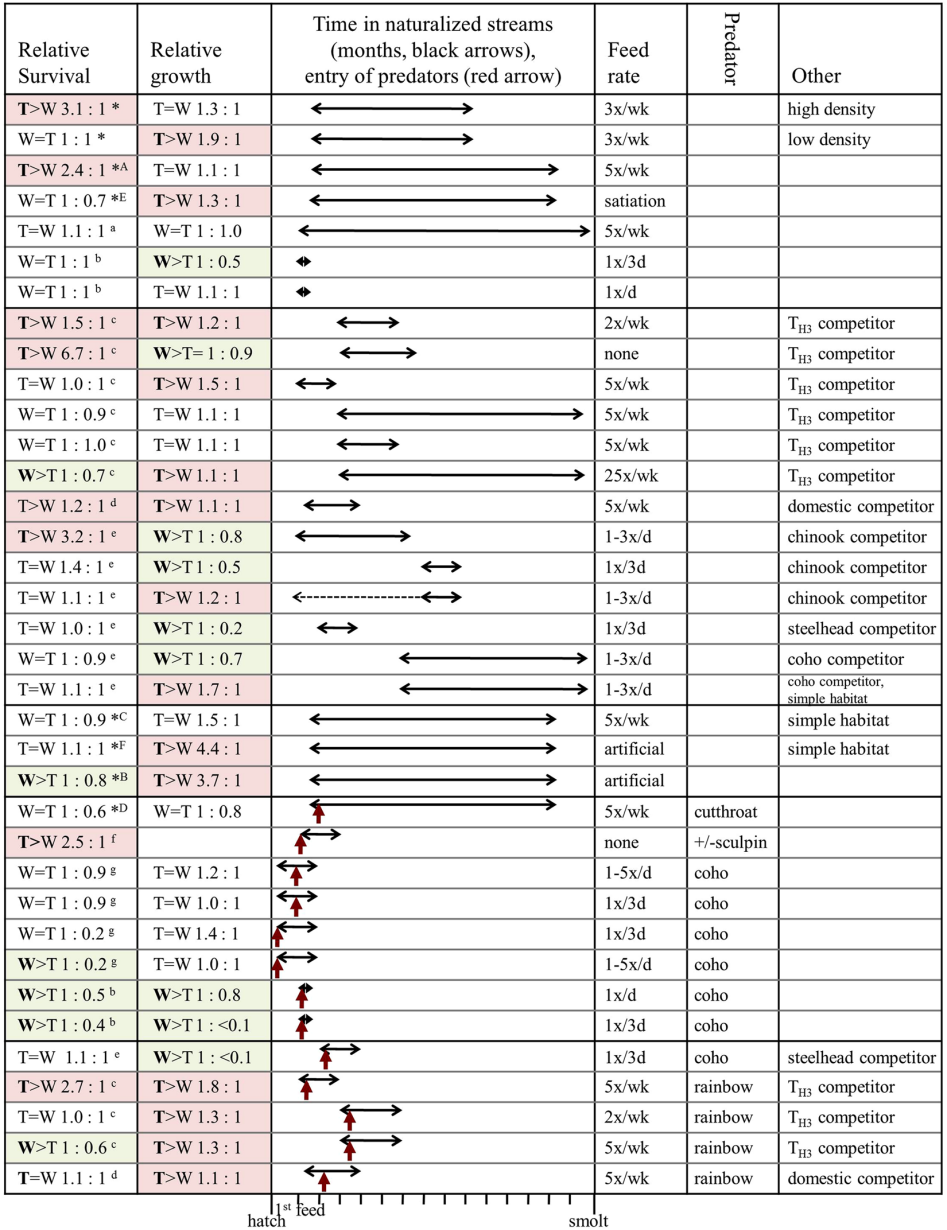
Summary of studies on survival and growth/size of M77-strain growth hormone transgenic (T) coho salmon juveniles relative to wild-type (W) in freshwater naturalized stream environments, with or without predators and/or competitors, under varying levels of supplemental natural food items. Horizontal lines indicate approximate time frame juvenile fish were in the stream. Red vertical arrow indicates time predators were introduced. Ratios of survival and growth are given relative to wild-type fish, red fill indicates indices significantly greater in T fish, and green fill indicates indices significantly greater in W fish. * indicates data is from current study, and superscripts indicate where data is from previously published studies: ^a^^27^; ^b^^26^; ^c^^29^; ^d^^13^; ^e^^23^; ^f^^21^; ^g^^25^.

The relative growth and survival of growth hormone (GH) transgenic and non-transgenic coho salmon has been found to be strongly influenced by environmental conditions. Under hatchery conditions, the transgenic fish consistently outgrew the non-transgenic fish in mass and length, however the growth of the two strains did not consistently differ if performance was assessed within semi-natural environments. Relative survival of one strain may be higher in one experiment, and lower in another. Given the high variation in results obtained from experiments seeking to assess the relative growth and survival of transgenic and wild-type fish in nature-like environments (see Fig. 4), it is important to understand the specific habitat factors that may be driving this variability. In the present experiments, we assessed the impacts of major variables affecting life history traits of salmonids in nature, namely density, food amount, food type, habitat complexity, and presence of a predator on the relative growth and survival of transgenic and non-transgenic coho salmon. From these results, food type (artificial feed versus natural feed) and rearing density had the greatest influence on the magnitude of difference in growth between the two types of fish.

Density was found to have a strong effect on relative survival and growth of transgenic versus non-transgenic fish. Under high-density rearing (60 fish per m^2^), all fish had reduced survival relative to low density (6 fish per m^2^) as has been observed in other salmonids in nature^34–36^, but the transgenic fish had higher survival than the non-transgenic fish regardless of food level. Estimates of maximum stream densities for stream dwelling salmon, based on literature review, are approximately 10–30 fish per m^2^ for fish with fork length of 4–6 cm; these estimates support our observation that our high density habitat would be expected to have impacts on population size or growth. Under these conditions, transgenic fish were less affected by a more competitive environment than non-transgenic fish (in this case, the fish are competing for food resources). Within the low density streams, there were no differences in survival. Mortality within the high-density streams was likely due to either cannibalism^13,37,38^ or starvation, suggesting that the food supplied to the stream was not sufficient to satiate all fish, even at the high food level (as normally is the case in nature; but see below for discussion of habitat complexity influences). Effects of food supply were further supported by the observations that the transgenic fish did not outgrow the non-transgenic fish under high-density conditions, and the overall size of both fish types was greatly reduced relative to low density rearing at both a high and low food level. While transgenic fish were not able to gain greater size under high density, under less competitive low-density conditions they grew larger than non-transgenic fish regardless of food level. This suggests transgenic fish are more likely to realize accelerated growth potential under conditions of low competition and other social interactions, and highlights the importance of considering density as an important factor in ecological risk assessments. To complicate this relationship, density impacts may be mediated through two different mechanisms depending on the level of density; as density increases, there can be a shift towards increased density-dependent mortality and reduced observations of density-dependent growth^35,39,40^.

In the experiment assessing effects of four habitat variables (habitat complexity, type of food, amount of food, and risk of predation), the transgenic fish greatly outgrew the non-transgenic fish under hatchery conditions as expected (e.g.^19^). Within stream tanks, when individual environmental components were altered to mimic those of hatchery conditions, only feeding of artificial food to satiation resulted in the same level of growth acceleration in transgenic fish reared in the hatchery. While feeding natural food to satiation did increase mass (but not length) of transgenic relative to non-transgenic fish, this difference was much lower than that observed when fed artificial food. This indicates transgenic fish reared as fry in natural conditions may not be able to realize full growth potential when exposed to natural food items, regardless of available quantity of those food items. In hatchery conditions, transgenic fish have altered enzymatic capacity to digest and utilize different food components, and are able to maintain accelerated growth even under artificial diets that are considered poor for non-transgenic fish^41^. Consequently, the lack of growth enhancement in transgenic fish when fed natural food may not be due to poor digestibility of energy levels of the natural diet. When fed an artificial diet, transgenic fish have been found to have greatly altered appetite signalling peptides in the brain regardless of food level^42^. It is possible that natural food items, and/or the requirement to forage for them, alter satiation and appetite signals in transgenic fish such that they are more similar to non-transgenic fish. Despite high growth of transgenic fish, they had lower survival than non-transgenic fish when fed an artificial diet, although this difference was not significant in simple stream habitats. Consequently, extreme accelerated growth may come at a survival cost to transgenic fish under differing environmental conditions and thus estimates of survival made in hatchery conditions with artificial foods may not accurately predict what may be observed in nature.

There were no differences in size between the genotypes in the stream tanks fed natural food items at a normal food level, and only minor differences under satiation conditions with natural food. Results under a natural food supply suggests that the habitats that received a lower feed ration more closely replicated the “high-density” treatment as categorized in the first experiment, whereas those fed to satiation more closely resembled the “low-density” treatment. Taken together, these results indicate growth advantage by transgenic fish may only be obtained under less-limiting conditions. In contrast, transgenic fish had lower mortality in the most limiting conditions in both experiments, indicating they may have better ability to acclimate to limiting conditions regardless of whether they can obtain a growth advantage. Sundström and Devlin^21^ also found intrinsic growth rates of transgenic fish did not strongly influence survival under nutritional stress.

The ability of fast-growing strains (e.g. GH transgenic or domesticated) to secure enough food for growth and survival under food-limited conditions has been observed in other studies^13,17,22,23,37,38,43–48^ and is thought to be associated with increased foraging and aggressive behaviour of the fast-growing fish. To maintain increased growth, transgenic fish must increase food intake and tend to show increased motivation to feed (see supplemental video in^5,49^), resulting in higher daily food intake and increased ability to compete for food relative to non-transgenic fish. The transgenic fish may also be more dominant and able to secure optimal positioning within the stream environment to minimize energy wasted and optimize food capture opportunities, which would support higher survival^50^. Previous work in naturalized streams found that transgenic salmon fry had different dispersal within the stream relative to non-transgenic fish^20,25^, however studies with GH transgenic Atlantic salmon did not find a difference in dominance in this species at first-feeding stage^28^.

Fish reared in hatchery tanks with an enriched environment, natural sources of food, and threat of predation can show a behavioural change towards a less risky foraging strategy compared to fish reared in bare tanks, even after only 10 days in the enriched environment^51^. Habitat enrichment has been shown to promote more natural behaviour in hatchery fish^52,53^ and improve the ability of hatchery-reared fish to forage on novel live prey^54^. Increasing the complexity of hatchery tanks can influence many aspects of fish biology, including aggression, stress, energy expenditure, injury and disease susceptibility^55^. However, within the present study, habitat complexity did not seem to have an impact on relative growth. It was expected that the simple stream environment should have had similar results as the typical hatchery environment since both environments were bare tanks with artificial food fed to satiation and no risk of predation. Under these conditions, it was expected that risky foraging behaviour would have been positively reinforced by successful acquisition of a food reward. However, this was not the case, perhaps due to differences in density. It is also possible that the difference in the means of food provision in a tank are affecting growth and survival; in round, deep culture tanks, fish can always rapidly detect incoming food resources at the water surface, whereas in the stream they may be in a position distant from the greatest feed density and either not notice the food or be unwilling to risk movement to it^50^. Within the hatchery culture environment, the density was 0.5500 fish per 1 L whereas in the simple stream tank density was ten-fold lower. The higher survival of the non-transgenic fish in the complex stream environment (with artificial feed to satiation and no risk of predation) suggests that the non-transgenic fish were better at hiding within the complex environment structures to avoid cannibalism from the faster-growing transgenic fish (e.g.^37^).

In summary, our data examine many of the influences on relative survival and growth of transgenic and non-transgenic coho salmon detected in previous experiments (Fig. 4), and critically examined their interactions. Depending on the environment, the transgenic fish can show growth rates that are higher than, equal to, or lower than those of the non-transgenic fish. Relative survival showed similar variation. Our study demonstrates that density of the fish within the habitat is an important factor driving relative growth and survival. Relative growth is most strongly influenced by the type of food; when fed manufactured fish feed designed to optimize salmonid growth, the transgenic fish outgrew the non-transgenic fish to a greater degree than in any other condition in the current or previous studies (see Fig. 4). Survival seemed to be more sensitive to the other environmental factors that we examined. Transgenic fish had higher survival within a complex environment with natural food fed at an amount less than satiation, but even when the amount was increased to satiation there was no difference in survival. If the food type was artificial instead of natural, then the non-transgenic fish showed increased survival relative to the faster-growing transgenic strain. Within a simple stream environment, transgenic and non-transgenic fish had similar survival regardless of food type (although this may be influenced by density, if the hatchery environment is compared with the simple stream tank). When the current and previous studies using naturalized stream tanks were grouped into general environmental conditions (e.g. presence/absence of predators/competitors, food rate, see Fig. 4) there was significant variation in relative success of transgenic versus non-transgenic juvenile coho salmon among and within environmental conditions. Under most environmental condition groups, there were some observations of transgenic fish performing equally as well as wild type. However, under conditions of natural food but no predators or additional competitors there was a trend towards transgenic fish performing better than wild type, while in the presence of predators there was a trend towards transgenic fish having lower performance than wild-type fish. There was no discernable trend when reared with wild or fast-growing (i.e. domesticated or alternate GH transgenic (T_H3_) strains) competitors in the absence of predators, but in the presence of predators and fast-growing competitors, transgenic fish generally performed better than wild type. While these general trends may help predict the performance of transgenic fish under specific environmental conditions, the large variation in results indicate we do not fully understand how environmental components interact with genotype to influence success in natural environments, and predictions on success would have significant uncertainty. It should also be noted that this experiment reflected results when the fish were released to the experimental conditions from a hatchery environment; if fish were exposed to enriched environments prior to the experiment, relative growth and survival could show different trends^20,23,51,56^.

## Conclusion

The experiments described here demonstrate that phenotypic plasticity leads to dynamic responses in relative growth and survival when tested under different environmental conditions. Despite the dynamic nature of the responses in growth and survival, it was consistent that a strong effect of transgenesis on relative fitness components (growth and survival) was apparent when the fish were fed a commercial diet designed to optimize salmonid growth, supporting other research that showed food source to be a driving factor in relative performance of transgenic and non-transgenic fish. Density also appears to have a major impact on relative growth and survival, but this is further complicated due to secondary effects of other environmental characteristics (such as food type and amount). The varied phenotypic responses of GH transgenic and wild-type coho salmon to environmental conditions is a consistent finding seen among previous studies, and illustrates the complexity of integrating these factors to allow accurate prediction of fitness and consequences of novel genotypes within the many complex conditions found in nature^4–6^. These data continue to highlight the difficulty in using laboratory-based experiments with limited ability to fully replicate nature to accurately predict risk in the wild. Generating risk conclusions from any single experiment or environmental condition in hatchery or simulated-nature environments in the laboratory has the potential to either overestimate or underestimate risk, and should be viewed with significant uncertainty unless effects of the genetic modification on fitness are so strong that environmental conditions do not influence their phenotype significantly. Although the focus here was on GH transgenic salmon, the results from this model system have relevance for risk assessments of other genetically modified strains, invasive species, and introduced wild-type and domesticated strains.
